# Rapid and Sensitive Determination of Timosaponin AIII in Rat Plasma by LC-MS/MS and Its Pharmacokinetic Application

**DOI:** 10.3390/ijms14023656

**Published:** 2013-02-07

**Authors:** Yanping Liu, Yiqiong Pu, Tong Zhang, Yue Ding, Bing Wang, Zhenzhen Cai

**Affiliations:** 1 School of Pharmacy, Shanghai University of Traditional Chinese Medicine, Shanghai 201203, China; E-Mail: lyp1066@hotmail.com; 2 Experiment Center for Teaching and Learning, Shanghai University of Traditional Chinese Medicine, Shanghai 201203, China; E-Mails: puyiq@hotmail.com (Y.P.); annabel_cn@163.com (B.W.); Czz2021@vip.sina.com (Z.C.)

**Keywords:** timosaponin AIII, LC-MS/MS, rat plasma, pharmacokinetic study

## Abstract

A rapid sensitive and selective liquid chromatography-tandem mass spectrometry (LC-MS/MS) method was developed for determination of timosaponin AIII (TA-III) in rat plasma, using ginsenoside Re as an internal standard (IS). TA-III and the IS were detected in MRM mode with a negative ionization electrospray mass spectrometer. The calibration curves were linear over the concentration ranges from 11.14 to 1114 ng/mL and the lower limit of quantification (LLOQ) was 11.14 ng/mL. Intra-day and inter-day precisions (RSD) were within 10%, and accuracy ranged from 6.4% to 9.1%. The extraction recovery at three concentrations ranged from 92.3% to 95.5%. The validated method was successfully applied to monitor the concentrations of TA-III in rat plasma after intragastric administration. The best fit pharmacokinetic model to estimate the pharmacokinetic parameters was a single compartment model with weight of 1/*x*^2^ for oral administration groups of rats for TA-III.

## 1. Introduction

Timosaponin AIII (TA-III) is a typical spirostanol saponin, originally isolated from the rhizome of *Anemarrhena asphodeloides*. It was reported that TA-III could ameliorate learning and memory deficits in mice [1]. Another studies showed that TA-III had remarkable anticancer activity [2,3] and anti-respiratory syncytial virus (RSV) properties [4], which were also revealed to be a pronounced activator of autophagy [5].

**Figure 1:**
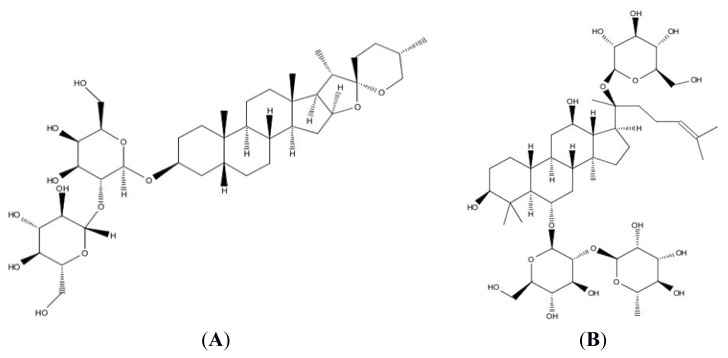
(**A**) Chemical structure of TA-III, TA-III MW = 740.92; and (**B**) ginsenoside Re (internal standard), ginsenoside Re MW = 945.4.

In view of its powerful pharmacological activities, it is very important to explore its pharmacokinetic behaviors, before which it is essential to develop a proper method to determine its concentration in plasma. There has been no report about the determination of TA-III in a biological matrix. In this paper, we aimed to establish a rapid, sensitive and selective liquid chromatography-tandem spectrometry (LC-MS/MS) method for the determination of TA-III in rat plasma. The analysis was achieved by using LC-MS/MS in negative ionization mode, and ginsenoside Re (IS) was employed as the internal standard (IS) [6]. The chemical structures of TA-III and ginsenoside Re were shown in Figure 1. The IS was found to have sensitive response, freed of interference with high recovery properties. TA-III and IS were identified and quantified by using multiple-reaction monitoring (MRM) mode, which enhanced the sensitivity and specificity of the analysis. The method was validated over the concentration range of 11.14–1140 ng/mL. The method was successfully applied to the pharmacokinetic study of TA-III after oral administration in rats. This is the detail research report on the pharmacokinetic study of TA-III, which may have a great significance for further research and application.

## 2. Experimental Section

### 2.1. Chemicals and Reagents

TA-III (Purity around 90%) for animal experiments was isolated and purified from the rhizome of *Anemarrhena asphodeloides* in our laboratory. TA-III (purity ≥ 98%) was purchased from Shanghai Yuanye Bio-Technology Co., Ltd., which was used as a reference standard compound for quantitative analysis of TA-III in biological samples. Its chemical structure was unambiguously identified by ESI-MS, ^1^H-NMR, and ^13^C-NMR spectra [7]. ^1^H-NMR(C_5_D_5_N) δ: 0.79(3H, s, 18-CH_3_), 0.95(3H, s, 19-CH_3_), 1.05(3H, d, J = 7.1 Hz, 27-CH_3_), 1.13(3H, d, J = 6.6 Hz, 21-CH_3_), 4.49(1H, d, J = 7.7 Hz, Gal 1-H), 5.27(1H, d, J = 7.7 Hz, GLC 1-H). ^13^C-NMR (DMSO) δ: 109.6, 106.1, 102.5, 81.8, 81.3, 78.3, 78.0, 76.9, 76.5, 75.4, 75.1, 71.6, 69.8, 65.0, 62.9, 62.7, 62.1, 56.4, 42.4, 40.8, 40.2, 40.1, 36.9, 35.4, 35.2, 32.1, 30.8, 30.8, 27.5, 26.9, 26.7, 26.7, 26.3, 26.1, 23.9, 21.0, 16.5, 16.2, 14.8. The internal standard (IS), ginsenoside Re (98% purity) was also purchased from the Shanghai Yuanye Bio-Technology Co., Ltd (Shanghai, China). Acetonitrile (HPLC grade) was obtained from Merck (Darmstadt, Germany). All other reagents were of analytical grade.

### 2.2. Instruments and Analytical Conditions

#### 2.2.1. Instruments

The analyses were performed on an Agilent 1290 Series high-performance liquid chromatography (HPLC) system (Agilent, USA), equipped with a G4220A binary pump, a G4226A autosampler and a G1316C thermosttated column compartment. An Agilent 6460A Triple Quad LC/MS equipped with an electrospray source was connected to the HPLC system. An Agilent Eclipse XDB-C_18_ column (50 mm × 2.0 mm, 1.8 μm) was used for liquid chromatographic separation.

#### 2.2.2. Analytical Conditions

The mobile phase was consisted of water and acetonitrile using gradient elution. The column was equilibrated and eluted under gradient conditions (shown in Table 1) with a flow rate of 0.4 mL/min, maintained at 25 °C. The sample injection volume was 5 μL.

**Figure 2:**
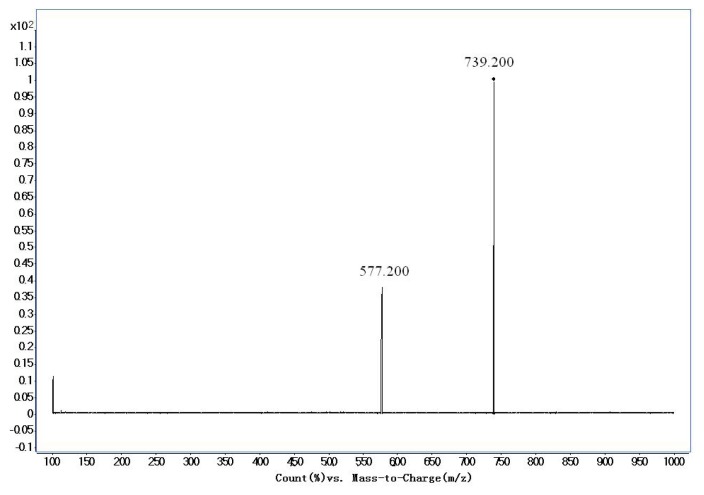
Production spectra of Timosapoin AIII.

**Figure 3:**
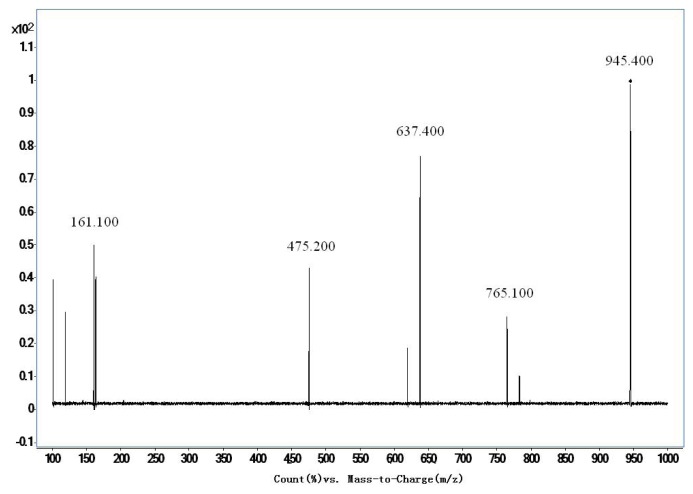
Production spectra of ginsenoside Re (IS).

The mass conditions of electrospray ionization were optimized as follows: Capillary −4500 V, gas temperature 350 °C, drying gas 10 L/min. Quantification was performed in negative multiple reaction monitoring (MRM). The optimized MRM parameters for TA-III and IS were shown in Table 2. Full scan product ion of precursor ions of TA-III and IS were shown in Figure 2 and 3.

### 2.3. Sample Preparation

A simple and rapid protein precipitation method was used for the preparation of plasma samples. 20 μL IS solution (7.4 μg/mL) and 300 μL methanol were added to 100 μL plasma sample. After vortexes for 5 min and centrifuged at 17,000× *g* for 10 min, all of the supernatant was transferred to a clean 1.5 mL centrifuge tube and evaporated to dryness under nitrogen. The obtained dried extract was reconstituted in 100 μL methanol by vortex-mixing for 5 min. Then the extracted sample was centrifuged at 19,000× *g* for 10 min. The supernatant was transferred into injector vials and a 5 μL aliquot was injected into LC-MS/MS system for analysis.

### 2.4. Animals and Pharmacokinetic Study

Male Sprague-Dawley rats (225 ± 25 g) were supplied by the Shanghai Experiment Animal Center and housed in an air-conditioned animal quarter at a temperature of 22–24 °C and the relative humidity of 50% ± 10%. They had free access to rodent chow and tap water prior to the experiments. Rats were fasted 12 h with free access to water prior to the test. Fifteen rats were randomly divided into three groups. An aqueous solution contained TA-III was intragastrically administrated to rats, respectively at doses of 25, 50, and 75 mg/kg, and the volume of administration was 1 mL/100 g. Blood samples (in each case approximately 0.3 mL), collected in heparinized tubes via the postorbital venous plexus veins from rats at 0.5, 1, 2, 4, 6, 7, 8, 10, 12, 14, 24, 27, 30, 36, 48 h after administration, were immediately centrifuged at 5000× *g* for 5 min to obtain the plasma. The samples were then pretreated with exactly the same procedure as described in Section 2.3. Plasma samples collected from 15 rats before administration were served as blank control samples. Animal experiments were carried out in accordance with the local institutional guidelines for animal care of Shanghai University of Traditional Chinese Medicine.

### 2.5. Method Validation

#### 2.5.1. Selectivity

The selectivity of the method was investigated by analyzing six individual blank plasma samples. The chromatographic findings of each control drug-free plasma containing neither analyte nor internal standard (double blank) were compared with the spiked rat plasma containing TA-III (557 ng/mL) and IS (1480 ng/mL) and the plasma sample was collected at 0.5 h after an oral dose of 50 mg/kg TA-III to check the absence of interference.

#### 2.5.2. Linearity and Lower Limit of Quantification (LLOQ)

The stock standard solutions of TA-III and IS were prepared by dissolving accurately weighed individual compounds in volumetric flasks with methanol to give a final concentration of 5.57 and 7.40 mg/mL, respectively. A series of standard working solutions at concentrations of 11.14–1140 ng/mL for TA-III were obtained by adequate dilution of the standard stock solution with methanol. IS working solution (7.40 ng/mL) was prepared by diluting its stock solution with methanol. All solutions were stored at 4 °C and brought to room temperature (20 °C) before use.

One hundred microliter of a series of standard solution of TA-III were transferred to a 1.5 mL centrifuge tube and evaporated to dryness under nitrogen. Then, 100 μL of blank plasma was added respectively, vortexed for 5 min, to obtain standard working solutions at concentrations of 11.14, 55.7, 111.4, 278.5, 557 and 1140 ng/mL. Then, the samples were pretreated with exactly the same procedure as described in Section 2.3. Each concentration level was prepared in six replicates. To evaluate linearity, plasma calibration curves were prepared and assayed in duplicate on six consecutive days over the range 11.4–1140 ng/mL for TA-III with the same concentration (1480 ng/mL) for IS. The contents of TA-III in the test samples were calculated using the regression parameters obtained from the standard curve. The acceptance criteria for a calibration curve were that each back-calculated standard concentration must be within 15% deviation from the nominal value except at the LLOQ, for which maximum acceptable deviation was set at 20%. The LLOQ was defined as the lowest concentration that gave a signal-to-noise ratio (S/N) of ≥10. The LOD demonstrated that the concentration that gave a signal-to-noise ratio (S/N) of 3.

#### 2.5.3. Precision and Accuracy

Three concentrations (11.14, 557 and 1114 ng/mL) of TA-III standard solutions were added to blank plasma to obtain quality control samples (QC Samples), respectively. Six replicates of each QC samples were prepared to determine on the same day for intra-day and on six consecutive days for the inter-day accuracy validation. The concentrations were calculated using calibration curves obtained on the day. The precision of the method at each QC concentration was expressed as the relative standard deviation (RSD) and the accuracy was described as relative error (RE), *i.e.*, (determined concentration-nominal concentration)/(nominal concentration) × 100%. The acceptance criteria for precision and accuracy were that RSD should be within 15% and the RE should be within 15% of the actual values for QC samples.

#### 2.5.4. Extraction Recovery

The three concentration-spiked (11.14, 557 and 1114 ng/mL) plasma samples containing TA-III were prepared, and then pretreated with exactly the same procedure as described in Section 2.3. Each level was pretreated in six replicates. At the same time, 100 μL standard solution of TA-III (11.14, 557 and 1114 ng/mL), together with 20 μL standard solution of IS (7.4 μg/mL), were respectively transferred to a 1.5 mL centrifuge tube, and evaporated to dryness under nitrogen. Then, 100 μL post-preparative blank plasma was added respectively to redissolve. Each concentration level was prepared in six replicates. The recoveries (extracted recovery) of TA-III from rat plasma after the extraction procedure were determined by comparing the peak areas of extracted TA-III or IS in spiked plasma sample with the area of TA-III or IS of the same concentration level dissolved in the post-preparative blank plasma (the final solution of blank plasma after extraction and dissolution with the initial mobile phase solution).

#### 2.5.5. Analyte Stability

Six replicates of QC samples (11.14, 557, 1114 ng/mL) were used to evaluate the stabilities of the analytes in rat plasma under the following storage conditions: post-preparative stabilities at room temperature for 24 h, stabilities of unpreparative stabilities at room temperature for 24 h, three freeze-thaw cycles and long-term stability storage at −20 °C for 50 days. Concentrations of TA-III in all samples were calculated by using freshly prepared calibration samples. The stability was described as relative error, *i.e.*, (determined concentration-nominal concentration)/(nominal concentration) × 100%. The RE should meet the following criteria: not more than 15% deviation for the three different concentration QC samples.

##### 2.5.5.1. Post-Preparative Stabilities at Room Temperature for 24 h

Three QC samples (11.14, 557, 1114 ng/mL) were pretreated with exactly the same procedure as described in Section 2.3, at room temperature (25 °C) for 24 h, to evaluate the post-preparative stabilities at room temperature. Each concentration level was prepared in six replicates.

##### 2.5.5.2. Stabilities of Unpreparative Sample at Room Temperature for 24 h

Three QC samples (11.14, 557, 1114 ng/mL) were placed at room temperature (25 °C) for 24 h, then pretreated with exactly the same procedure as described in Section 2.3, to evaluate the stabilities of unpreparative sample at room temperature. Each concentration level was prepared in six replicates.

##### 2.5.5.3. Three of Freeze-Thaw Cycles

Three QC samples (11.14, 557, 1114 ng/mL) were detected after the three freeze–thaw cycles at −20 °C. Each concentration level was prepared in five replicates.

##### 2.5.5.4. Long-Term Stability Storage at −20 °C for 50 days

Three QC samples (11.14, 557, 1114 ng/mL) were stored at −20 °C, detected on the first and 50th day. Each concentration level was prepared in five replicates.

#### 2.5.6. Matrix Effect

To evaluate the absolute matrix effect on the ionization of TA-III and IS, the peak areas of the compounds dissolved in the blank samples with three concentrations of TA-III (11.14, 557, 1114 ng/mL) and IS (1480 ng/mL) were compared with those of the compounds only dissolved in methanol. The corresponding peak areas of TA-III or IS in spiked plasma post-extraction (A) were then compared with those of the solution standards in methanol (B) at equivalent concentrations. The ratio (A/B × 100%) is defined as the absolute matrix effect.

### 2.6. Statistical Analysis

To determine the pharmacokinetic parameters of TA-III, the concentration-time data were analyzed by DAS Software (version 2.0, China State Drug Administration). Data was expressed as means ± SD.

## 3. Method Validation

### 3.1. Selectivity

**Figure 4:**
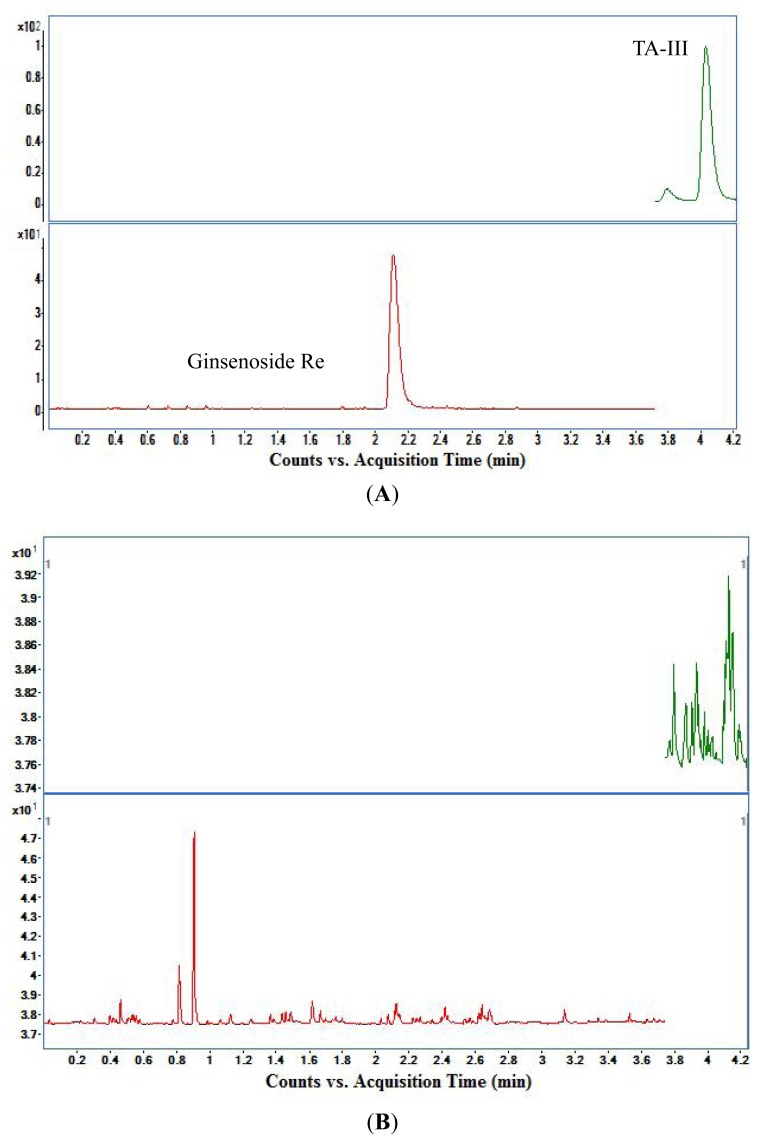

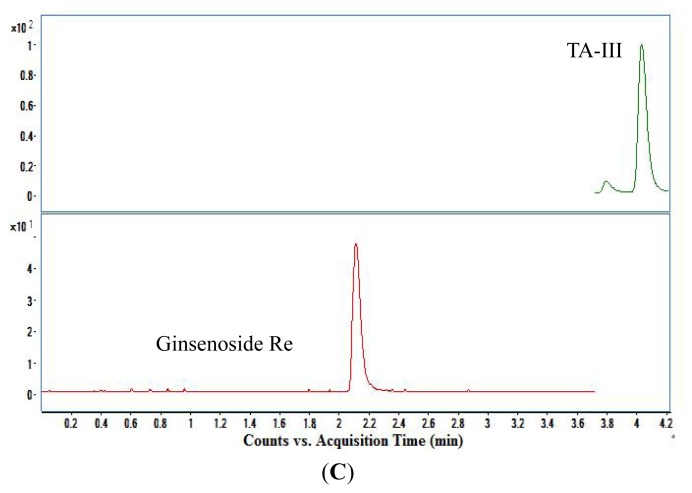
Representative MRM chromatograms of TA-III and IS for (**A**) a plasma sample from a rat subject collected at 0.5 h after an oral dose of 50 mg/kg TA-III; (**B**) a blank rat plasma; (**C**) a spiked sample at the concentration of 557.0 ng/mL for TA-III and 1480 ng/mL for IS.

The representative MRM chromatograms of plasma sample collected at 0.5 h after an oral dose of 50 mg/kg TA-III, blank rat plasma and spiked plasma (*n* = 6) were shown in Figure 4. The retention time was about 4.14 min for TA-III and 2.16 min for ginsenoside Re (IS). As shown in Figure 4, no interfering peaks were observed in the representative chromatogram of blank plasma at the retention time of TA-III or IS.

### 3.2. Linearity and Lower Limit of Quantification (LLOQ)

The calibration curve was obtained from the peak-area ratios of each analyte to IS *versus* plasma concentrations using a 1/*x*^2^ weighted linear least-squares regression model. The calibration curve for spiked rat plasma of TA-III was *y* = 0.0021*x* + 0.1111 (*r* = 0.9960, *n* = 6), linear over the range 11.14–1114 ng/mL, with a correlation coefficient *r*^2^ > 0.99. The date was shown in Table 3. The lower limit of quantification (LLOQ) of TA-III in plasma was 11.14 ng/mL.

### 3.3. Precision and Accuracy

The results of intra- and inter-day precisions and accuracies at three concentrations of TA-III were listed in Tables 4, 5 and 6. The intra-day precisions ranged from 3.4% to 7.8%, while the inter-day precisions ranged from 2.7% to 3.5%. Accuracy was determined as the percentage difference between the mean concentrations detected and the nominal concentrations. The accuracy derived from QC samples ranged from 6.4% to 9.1%. The results demonstrated that this method had satisfactory accuracy and precision.

### 3.4. Extraction Recovery

Extraction recovery was determined at three QC levels (11.14, 557 and 1114 ng/mL) by comparing peak areas obtained from plasma samples with those obtained by direct assay of a standard solution with the same concentration. The results were shown in Table 7. The extraction recovery of TA-III at three different concentrations ranged from 92.3% to 95.5%. These statistic data indicated that the extent of recovery of TA-III and IS was consistent, precise and reproducible.

### 3.5. Analyte Stability

Concentrations of TA-III in all samples were analyzed by using freshly prepared calibration samples. The stability was described as relative error, *i.e.*, (determined concentration-nominal concentration)/(nominal concentration) × 100%. The results met the following criteria: not more than 15% deviation for the three different concentration QC samples. The stability data were shown in Tables 8, 9, 10 and 11. It indicated that the three analytes in plasma were stable under a variety of storage conditions: post-preparative and unpreparative at room temperature for 24 h, at −20 °C for 50 days and three of freeze–thaw cycles, with the average deviations being within 15% of the nominal values.

### 3.6. Matrix Effect

The peak area of the post-extraction blank plasma spiked with standard solution of TA-III and IS was known as set 1, and the peak area of the diluted standard solutions in methanol at the same concentration was known as set 2. Calculate the peak-area ratio of the analytes in set 1 with that in set 2. The results are shown in Table 12. The matrix effects were no less than 85% or more than 115% for all the five analytes, which showed no significant difference between the peak areas of samples prepared from rat plasma and standard solution. It indicated that the matrix effect had no significant influence on the determination of TA-III in rat plasma.

## 4. Pharmacokinetic Analysis

**Figure 5:**
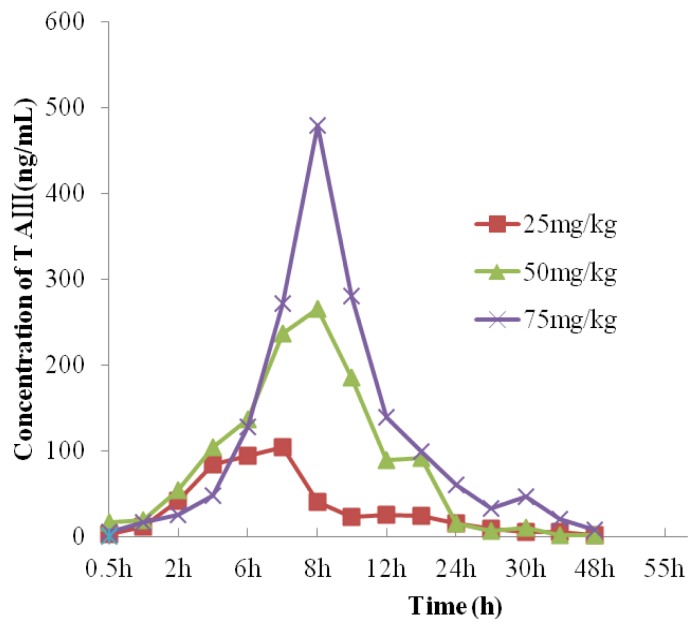
Plasma concentration-time curves of TA-III after intragastric administration to rats at dose of 25, 50 and 75 mg/kg.

The results of the concentration time at the three doses of TA-III were shown in Tables 13, 14 and 15. The mean plasma concentration-time curves were illustrated in Figure 5. The pharmacokinetic parameters were estimated by DAS software. The corresponding pharmacokinetic parameters were listed in Tables 16–19.

## 5. Conclusions

The LC-MS/MS method for determining TA-III in biological samples was in accordance with the guidance on bioanalysis criteria in methodological investigation. The method had been successfully used for pharmacokinetic studies of TA-III with intragastrical administration in rats. The concentration of TA-III for up to 48 h after administration of TA-III could be detected for all groups with different doses. In the statistical process, it was found that the best fit pharmacokinetic model to estimate the pharmacokinetic parameters was a single compartment model with the weight of 1/*x*^2^ for oral administration groups of rats by using DAS 2.0 software. The results showed that C (max) of TA-III was mainly dependent on the dose with the coefficient of correlation of 0.9988 in the three groups. AUC of TA-III has a bad relationship with the dose with the coefficient of a correlation of 0.9166. The half-lives (*t*_1/2_) were 1.55 ± 0.546, 2.60 ± 0.56 and 1.382 ± 0.420 h, which demonstrated that the elimination of TA-III was relatively quick. Mean residence time (MRT) was 12.1 ± 4.8, 11.0 ± 1.0 and 16.2 ± 2.8 h, thereby revealing the body retention time of this compound was long, and indicating, in turn, that TA-III was difficult to be absorbed, but easy to be eliminated in rats. These pharmacokinetic properties will have inevitable influences on its biological effects.

## Figures and Tables

**Table 1:** HPLC-MS/MS mobile phase gradient elution condition.

Time (min)	A (H_2_O)	D (Acetonitrile)	Flow (mL/min)
0	85%	15%	0.4
0.5	85%	15%	0.4
6.0	10%	90%	0.4
7.0	10%	90%	0.4
7.1	85%	15%	0.4
9	85%	15%	0.4

**Table 2:** Optimized multiple-reaction monitoring (MRM) parameters for Timosapoin AIII and ginsenoside Re (IS).

Sample Name	Precursor/Product Ion	Dwell (msec)	Fragmentor	CE
Timosapoin AIII	739.2/577.2	200	260	30
Ginsenoside Re	945.4/475.2	200	280	60

**Table 3:** Calibration curve of TA-III.

Concentrations (ng/mL)	11.14	55.7	111.4	278.5	557	1114
Peak-area ratios (*n* = 6)	0.0526	0.2105	0.4075	0.7726	1.2768	2.4608
Calibration curve	*y* = 0.0021*x* + 0.1111(*r* = 0.9960, *n* = 6)					

**Table 4:** Intra-day precision for the analysis of TA-III (*n* = 6).

Concentration (ng/mL)	1	2	3	4	5	6	AVE	RSD (%)
11.14	10.5	10.7	10.9	10.9	11.4	12.9	11.2	7.8
557	593.3	554.8	580.6	602.4	558.5	562.8	575.4	3.4
1114	1143.3	1137.0	1108.6	1101.0	1036.9	1065.0	1098.0	3.8

**Table 5:** Inter-day precision for the analysis of TA-III (*n* = 6).

Concentration (ng/mL)	1d	2d	3d	4d	5d	6d	AVE	RSD (%)
11.14	11.2	11.1	11.1	11.1	11.0	11.9	11.2	3.0
557	575.4	536.5	571.4	577.9	561.8	566.9	566.5	2.7
1114	1098.0	1081.4	1085.4	1072.7	1042.3	1166.0	1092.0	3.5

**Table 6:** Accuracy for the analysis of TA-III (*n* = 6).

Concentration (ng/mL)	RE (%)						AVE (%)

	1	2	3	4	5	6	
11.14	6.4	12.2	11.3	4.3	4.1	11.5	9.1
557	5.8	5.0	1.3	9.2	10.9	11.4	7.3
1114	4.8	11.3	10.4	0.3	6.6	5.0	6.4

**Table 7:** Extraction recovery of TA-III from spiked rat plasma (n = 6).

Sample	Extract recovery (%)						AVE (%)	RSD (%)

Concentration (ng/mL)								
11.14	93.6	93.8	99.6	96.1	89.5	88.4	93.5	3.9
557	83.2	93.2	92.5	94.4	94.0	96.4	92.3	5.0
1114	95.6	97.5	97.1	97.2	96.2	89.6	95.5	1.2

**Table 8:** Post-preparative stabilities at room temperature for 24 h (*n* = 6).

Concentration (ng/mL)	1	2	3	4	5	6	AVE	RE (%)
11.14	10.8	11.4	11.4	11.1	11.5	11.2	11.2	0.8
557	600.7	592.4	603.7	588.7	621.9	566.9	595.7	6.9
1114	1135.0	1153.7	1076.2	1062.6	1152.3	1081.9	1110.3	3.3

**Table 9:** Unpreparative stabilities at room temperature for 24 h (*n* = 6).

Concentration (ng/mL)	1	2	3	4	5	6	AVE	RE (%)
11.14	10.2	10.6	11.4	11.7	11.2	11.7	11.1	0.2
557	582.4	512.3	582.0	583.8	559.5	580.7	566.8	1.7
1114	1149.1	1076.6	1066.7	1131.9	1073.3	1070.2	1094.6	1.7

**Table 10:** Stabilities of three of freeze-thaw cycles (*n* = 6).

Concentration (ng/mL)	1	2	3	4	5	6	AVE	RE (%)
11.14	10.9	10.6	11.0	10.6	10.9	12.1	11.0	1.0
557	560.4	547.0	547.2	524.5	553.6	563.7	549.4	1.4
1114	969.8	1091.1	984.6	1007.6	1018.7	996.3	1011.4	9.2

**Table 11:** Long-term stability storage at −20 °C for 50 days (*n* = 6).

Concentration (ng/mL)	1 day	50 days	AVE	RE (%)
11.14	11.9	11.2	11.6	3.3
557	566.9	564.3	565.6	1.5
1114	1166.0	1096.9	1131.5	1.6

**Table 12:** Matrix effect of TA-III in rat plasma (*n* = 5).

Sample	Matrix Effect of TA-III (%)						
Concentration (ng/mL)	1	2	3	4	5	AVE (%)	RSD (%)
11.14	112.8	106.1	106.0	110.4	104.0	107.9	3.4
557	110.2	107.8	92.2	113.6	115.0	107.8	8.5
1114	105.3	109.8	99.3	97.7	97.5	101.9	5.3

**Table 13:** Concentration time of TA-III after intragastrical administration to rats at a dose of 25 mg/kg (ng/mL).

Time (h)	Rat 1	Rat 2	Rat 3	Rat 4	Rat 5	AVE	SD
0.5	3.7 *	0.0	0.4 *	1.5 *	12.2	3.5 *	1.42
1	17.2	3.1 *	6.1 *	13.7	17.8	11.6	0.57
2	42.0	58.9	18.6	47.0	44.7	42.2	0.35
4	95.0	90.5	54.1	82.6	98.6	84.2	0.21
6	95.4	97.5	62.7	127.2	86.9	93.9	0.25
7	100.7	91.3	93.2	131.0	106.3	104.5	0.15
8	37.7	32.7	28.4	89.0	16.7	40.9	0.68
10	33.9	22.8	25.9	25.3	9.7 *	23.5	0.37
12	18.4	13.1	42.3	39.4	11.0 *	24.8	0.60
14	12.3	9.7 *	47.4	34.5	16.4	24.1	0.67
24	6.6 *	8.4 *	31.8	21.7	11.1 *	15.9	0.67
27	3.5 *	3.9 *	24.5	13.1	1.5 *	9.3 *	1.03
30	5.9 *	3.8 *	11.6	4.1 *	0.2 *	5.1 *	0.81
36	1.9 *	0.5 *	13.0	11.3 *	0.5 *	5.4 *	1.14
48	0.2 *	1.4 *	7.7 *	1.9 *	0.0 *	2.2 *	1.40

* Represents the detected concentrations below LLOQ.

**Table 14:** Concentration time of TA-III after intragastrical administration to rats at a dose of 50 mg/kg (ng/mL).

Time (h)	Rat 1	Rat 2	Rat 3	Rat 4	Rat 5	AVE	SD
0.5	5.9 *	27.6	17.8	17.1	12.7	16.2	0.49
1	6.6 *	29.3	37.2	11.8	10.7 *	19.1	0.70
2	12.6	63.1	94.5	85.0	15.2	54.1	0.71
4	29.5	122.2	129.7	183.8	53.3	103.7	0.60
6	45.7	129.5	237.4	148.0	122.3	136.6	0.50
7	71.4	237.4	340.8	401.1	131.3	236.4	0.58
8	109.6	285.6	285.5	381.8	261.3	264.8	0.37
10	234.0	199.9	226.5	88.0	181.2	185.9	0.32
12	55.0	44.9	108.2	104.4	131.6	88.8	0.42
14	69.1	84.7	87.9	110.4	107.5	91.9	0.19
24	1.0 *	51.1	5.9 *	10.9 *	6.3 *	15.0	1.36
27	11.6	10.6 *	0.0	2.7 *	6.2 *	6.2 *	0.80
30	13.6	11.0 *	11.0 *	5.8 *	9.6 *	10.2 *	0.28
36	3.5 *	1.8 *	1.8 *	0.0	3.2 *	2.0 *	0.68
48	0.4 *	2.3 *	1.7 *	1.6 *	0.0	1.2 *	0.81

* Represents the detected concentrations below LLOQ.

**Table 15:** Concentration time of TA-III after intragastrical administration to rats at a dose of 75 mg/kg (ng/mL).

Time (h)	Rat1	Rat 2	Rat 3	Rat 4	Rat 5	AVE	SD
0.5	0.0	26.3	0.0	0.0	0.0	5.3 *	2.24
1	30.1	38.2	2.6 *	9.4 *	2.8 *	16.6	0.99
2	15.1	38.2	11.6	42.0	20.2	25.4	0.54
4	54.6	63.2	8.0	61.1	51.9	47.8	0.47
6	170.1	71.7	48.9	274.2	71.9	127.3	0.74
7	304.2	180.0	104.3	357.5	414.1	272.1	0.47
8	546.1	298.7	363.0	607.3	583.3	479.7	0.29
10	300.8	305.0	196.9	350.9	247.3	280.2	0.21
12	141.6	156.4	117.1	181.0	99.1	139.0	0.23
14	121.1	104.9	121.9	88.9	60.7	99.5	0.26
24	51.8	67.0	22.1	59.8	103.5	60.8	0.48
27	37.9	31.5	19.8	50.5	22.2	32.4	0.38
30	49.2	142.5	6.4 *	13.1	22.6	46.8	1.20
36	12.7 *	12.7	26.9	16.3	31.5	20.0	0.43
48	5.6 *	6.8 *	8.8 *	3.0 *	12.5	7.3 *	0.49

* Represents the detected concentrations below LLOQ.

**Table 16:** Pharmacokinetic parameters of TA-III after intragastrical administration to rats at a dose of 25 mg/kg.

Parameters	Rat1	Rat 2	Rat 3	Rat 4	Rat 5	MEAN ± SD
T (peak)(h)	7	6	7	7	7	6.8 ± 0.4
C (max)(ng/mL)	100.7	97.5	93.2	131	106.3	105.7 ± 14.9
Ke (1/h)	0.352	0.374	0.051	0.274	0.419	0.294 ± 0.146
Ka (1/h)	0.407	0.416	0.984	0.314	0.472	0.519 ± 0.166
t_1/2_ (ka)(h)	1.703	1.665	0.704	2.209	1.47	1.55 ± 0.546
t_1/2_ (h)	1.969	1.854	5.693	2.528	1.653	2.74 ± 1.68
MRT (0–∞)(h)	9.35	9.051	19.842	13.592	8.421	12.1 ± 4.8
AUC (0–∞)[(ng/mL)h]	766.1	729.6	1347.2	1093.9	672.1	921.8 ± 289.0

**Table 17:** Pharmacokinetic parameters of TA-III after intragastrical administration to rats at a dose of 50 mg/kg.

Parameters	Rat1	Rat 2	Rat 3	Rat 4	Rat 5	MEAN ± SD
T (peak)(h)	10	8	7	7	8	8 ± 1.2
C (max)(ng/mL)	234	285.6	340.8	401.1	261.3	304.6 ± 66.8
ke (1/h)	0.279	0.195	0.205	0.3	0.209	0.238 ± 0.048
ka (1/h)	0.343	0.218	0.238	0.354	0.238	0.278 ± 0.065
t_1/2_ (ka)(h)	2.022	3.175	2.912	1.958	2.91	2.60 ± 0.56
t_1/2_ (h)	2.482	3.556	3.38	2.313	3.311	3.01 ± 0.57
MRT (0–∞)(h)	12.226	11.663	9.877	10.122	11.304	11.038 ± 1.007
AUC (0–∞)[(ng/mL)h]	1395.4	2682.6	3300.0	2694.1	2347.9	2484.0 ± 698.8

**Table 18:** Pharmacokinetic parameters of TA-III after intragastrical administration to rats at a dose of 75 mg/kg.

Parameters	Rat1	Rat 2	Rat 3	Rat 4	Rat 5	MEAN ± SD
T (peak)(h)	8	10	8	8	8	8.4 ± 0.9
C (max)(ng/mL)	546.1	305	363	607.3	583.3	480.9 ± 137.4
Ke (1/h)	0.386	0.088	0.313	0.364	0.548	0.340 ± 0.166
Ka (1/h)	0.567	0.332	0.496	0.571	0.7	0.533 ± 0.134
t_1/2_ (ka)(h)	1.222	2.088	1.396	1.213	0.991	1.382 ± 0.420
t_1/2_ (h)	1.794	7.908	2.216	1.902	1.264	3.017 ± 2.756
MRT (0–∞)(h)	14.236	17.46	19.741	12.753	16.889	16.216 ± 2.754
AUC (0–∞)[(ng/mL)h]	2938.3	4081.9	1992.8	3432.8	2424.3	2974.07 ± 822.2

**Table 19:** Pharmacokinetic parameters of TA-III after intragastrical administration to rats at different dose.

Parameters	25 mg/kg	50 mg/kg	75 mg/kg
Ke (1/h)	0.294 ± 0.146	0.238 ± 0.048	0.340 ± 0.166
Ka (1/h)	0.519 ± 0.166	0.278 ± 0.065	0.642 ± 1.393
t_1/2_ (ka)(h)	1.55 ± 0.546	2.60 ± 0.56	1.382 ± 0.420
t_1/2_ (h)	2.74 ± 1.68	3.01 ± 0.57	3.02 ± 2.76
T (peak)(h)	6.8 ± 0.4	8 ± 1.2	8.4 ± 0.9
C (max)(ng/mL)	105.7 ± 14.9	304.6 ± 66.8	480.9 ± 137.4
AUC [(ng/mL)h]	921.8 ± 289.0	2484.0 ± 698.8	2974.07 ± 822.2
MRT (h)	12.1 ± 4.8	11.0 ± 1.0	16.2 ± 2.8
